# A Low FODMAP Gluten-Free Diet Improves Functional Gastrointestinal Disorders and Overall Mental Health of Celiac Disease Patients: A Randomized Controlled Trial

**DOI:** 10.3390/nu10081023

**Published:** 2018-08-04

**Authors:** Leda Roncoroni, Karla A. Bascuñán, Luisa Doneda, Alice Scricciolo, Vincenza Lombardo, Federica Branchi, Francesca Ferretti, Bernardo Dell’Osso, Valeria Montanari, Maria Teresa Bardella, Luca Elli

**Affiliations:** 1 Centre for the Prevention and Diagnosis of Celiac Disease/Division of Gastroenterology and Endoscopy, Fondazione IRCCS Ca’ Granda Ospedale Maggiore Policlinico, 20122 Milan, Italy; kbascunan@med.uchile.cl (K.A.B.); scricciolo.alice@gmail.com (A.S.); vicky.l@hotmail.it (V.L.); federica.branchi@gmail.com (F.B.); francesca.ferretti01@gmail.com (F.F.); montanari.valeria8@gmail.com (V.M.); mariateresa.bardella@yahoo.com (M.T.B.); luca.elli@policlinico.mi.it (L.E.); 2 Department of Biomedical, Surgical and Dental Sciences, University of Milano, 20100 Milan, Italy; luisa.doneda@unimi.it; 3 Department of Nutrition, University of Chile, 8380453 Santiago, Chile; 4 Department of Pathophysiology and Transplantation, Università degli Studi di Milano, Fondazione IRCCS Ca’ Granda, Ospedale Maggiore Policlinico, 20100 Milan, Italy; bernardo.dellosso@unimi.it; 5 Department of Psychiatry and Behavioral Sciences, Bipolar Disorders Clinic, Stanford University, Stanford, CA 94305-5723, USA; 6 CRC “Aldo Ravelli” for Neuro-technology & Experimental Brain Therapeutics, University of Milan, 20100 Milan, Italy

**Keywords:** gluten-free diet, gastrointestinal symptoms, quality of life

## Abstract

A subset of patients with celiac disease (CD) on a gluten-free diet (GFD) reported the persistence of functional gastrointestinal disorders. Foods containing fermentable, oligosaccharides, disaccharides, monosaccharides, and polyols (FODMAP) can trigger a broad range of gastrointestinal symptoms in sensitive individuals. We evaluated the effects of a low FODMAP diet (LFD) on gastrointestinal and psychological symptomatology in CD patients. A total of 50 celiac patients on GFDs and with persistence of gastrointestinal symptoms were included. The patients were randomly allocated to one of two dietary groups—one on a low FODMAP GFD (LF-GFD, *n* = 25) and the other on a regular GFD (R-GFD, *n* = 25)—for 21 days. Psychological symptomatology and quality of life were evaluated by the Symptom Checklist-90-R (SCL-90) and the Short Form (36) Health Survey (SF-36) questionnaires, respectively. Gastrointestinal symptomatology and general well-being were evaluated by visual analogue scale (VAS) scores. After 21 days, 21 and 23 patients completed the dietary treatment on LF-GFD and R-GFD, respectively. A reduced global SCL-90 index (*p* < 0.0003) was found in the LF-GFD group but not in the R-GFD one. However, the SF-36 scores did not differ between groups after treatment. The VAS for abdominal pain was much lower, and the VAS for fecal consistency enhanced after treatment in the LF-GFD group. General well-being increased in both groups but with a much higher improvement in the LF-GFD (*p* = 0.03). A short-term LFD regimen helps to improve the psychological health and gastrointestinal symptomatology with enhanced well-being of CD patients with persisting functional gastrointestinal symptomatology. The long-term clinical effects of LFD in particular subgroups of CD patients need further evaluation.

## 1. Introduction

Celiac disease (CD) is an autoimmune multisystem disorder triggered by gluten ingestion [1,2]. CD affects genetically susceptible individuals who are known to possess the Human Leukocyte Antigen HLA DQ2 (90%–95%) or the HLA DQ8 (5%–10%) haplotypes [3]. CD symptomatology is mainly gastrointestinal with patients usually reporting diarrhea, bloating, abdominal pain, and weight loss [2]. Extra-intestinal symptoms can be also frequent [4].

A gluten-free diet (GFD) is the current treatment for CD [5]. In this dietary treatment, foods containing gluten, which is a protein found in grains, such as wheat, barley, rye, and triticale, are excluded. Gluten induces small intestine inflammation, and a GFD helps to counteract the clinical signs/symptoms and to prevent complications [6]. Although this treatment is highly successful, following a strict GFD poses great difficulty to patients in their family, social, and working contexts, thus deteriorating their quality of life [7] and causing psychological distress [8].

It is not uncommon that patients on GFDs report symptoms resembling those of irritable bowel syndrome (IBS), which is a frequent condition in clinical practice. Reportedly, around 20–23% of treated CD patients fulfill the Rome III criteria for IBS and also suffer from various functional gastrointestinal symptoms, further affecting their quality of life [9]. In fact, a meta-analysis has shown that IBS-like symptoms are common in CD patients (the pooled prevalence of IBS symptoms in treated CD patients was 38%), concluding that higher levels of adherence to a GFD are possibly associated with some reduction in symptomatology [10]; however, the authors also highlighted that in some patients, IBS-like symptoms persist even after following a strict GFD.

Functional gastrointestinal disorders are characterized by recurrent or current gastrointestinal symptoms that have no identifiable structural or biochemical basis. The most common functional gastrointestinal disorder is IBS [11]. The variety of clinical manifestations has limited the effective treatment of these syndromes, and most treatments to date only alleviate the primary manifestation. A novel option for IBS treatment, which is currently generating great excitement, is the dietary regimen with reduced amounts of fermentable, oligosaccharides, disaccharides, monosaccharides, and polyols (FODMAP) [12]. FODMAP are short-chain carbohydrates that are poorly absorbed in the small intestine and increase gas production and intestinal osmolarity because of their rapid fermentation and osmotic action [13,14,15]. Foods containing FODMAP can trigger gastrointestinal symptoms in sensitive individuals [13,16].

A low FODMAP diet (LFD) appears to be associated with the reduction of IBS symptoms [17]. A very recent meta-analysis found evidence for the short-term efficacy and safety of LFD for patients with IBS, but the long-term effects are still under investigation [18]. Recently, LFD has been evaluated in patients with inflammatory bowel disease, showing improved symptomatology after treatment [19]. From a clinical point of view, CD and IBS may coexist [20]. However, it is more likely that the inflammatory process occurring in CD does not revert completely in some patients on GFD, and similar low-grade inflammation can be present both in patients with CD and IBS [9,21]. To date there are no reports showing the potential effect of LFD on gastrointestinal symptomatology for patients with CD; thus, we have evaluated the role of LFD on treated CD patients with the persistence of functional gastrointestinal disorders. In addition, given the frequent manifestation of psychopathological and behavioral abnormalities in CD patients undergoing dietary changes, their overall psychological distress and disability were also assessed. In particular, we hypothesized that patients being administered LFD would show improved conditions in terms of gastrointestinal and psychopathological symptoms compared with patients on regular-GFD (R-GFD).

## 2. Materials and Methods 

This was a randomized double-blind intervention-controlled study, previously registered at ClinicalTrials.gov (ref. no. IDNCT02946827). All the authors had access to the study data and reviewed and approved the final version of the manuscript. The patients were recruited at the Center for Prevention and Diagnosis of Celiac Disease of Fondazione IRCCS Ca’ Granda Ospedale Maggiore Policlinico in Milan (Italy). The University of Milan’s Institutional Review Board reviewed and approved the study protocol according to the Helsinki Declaration, the Project Identification Code of the Ethics Committee Approval of our study: 744_2015bis, the protocol was approved by the Ethics Committee of Milano Area B (date 10.11.2015). All the patients gave and signed their informed consent prior to participation in this study. 

Between December 2015 and December 2017, we studied patients with CD fulfilling the following inclusion criteria: adult age (between 18 and 60 years), treated with a GFD for at least one year, with negative plasma tissue transglutaminase values, with IBS-like symptoms and functional gastrointestinal disorders according to the Rome III criteria [22], and with a global well-being score assessed by a visual analogue scale (VAS) of <4. As exclusion criteria, we considered the following: low adherence to the GFD (as evaluated by the Celiac Dietary Adherence Test [23]), refractory CD (as evaluated through biopsy to assess the persistence of intestinal atrophy while on a GFD and by means of interview carried out by a trained nutritionist, who assessed patients’ adherence to the diet), individual intolerance to disaccharides (as evaluated by hydrogen test (lactose and fructose), history of previous nutritionist evaluation or nutritional treatment for the dietary management of IBS, taking IBS pharmacological therapy, abdominal surgery, and type 2-diabetes. 

CD was diagnosed according to positivity to the serological tests of endomysial antibodies and tissue transglutaminase antibodies and on the basis of histological abnormalities at duodenal biopsy according to the modified Marsh classification (following the European Society for Pediatric Gastroenterology and Nutrition criteria) [24]. The allocation ratio was 1:1, and the recruited patients (*n* = 50) were randomly allocated to either of two dietary treatments—a low FODMAP GFD (LF-GFD, *n* = 25) or a R-GFD (R-GFD, *n* = 25)—for a time length of 21 days. Before randomization (baseline) and at day 21, the patients underwent a physical examination, and biochemical and nutritional parameters were assessed. After the intervention period, 21 patients in the LF-GFD and 23 in the R-GFD group completed the protocol and were included for the analyses reported in this study. The primary outcome was change in the VAS score for general well-being after 21 days of intervention. Secondary outcomes were changes in the VAS score for gastrointestinal symptomatology and the Short Form (36) Health Survey (SF-36) and the Symptom Checklist-90-R (SCL-90) scores for quality of life and psychological symptomatology, respectively.

The sample size was calculated using G*Power v. 3.1.9.2 for Windows (Düsseldorf, Germany [25]) based on the difference in the reduction of overall IBS-like symptomatology of at least 50% after 21 days following a LFD or high-FODMAP diet in IBS patients, as reported by McIntosh et al. [26]. Considering an α-error 1% (two-tailed test) and a power of 90%, with a response of 72% after the LFD and 21% after the high-FODMAP diet, the estimated sample size was estimated in 22 patients per group, including an additional 20% of patients for potential losses during the follow-up. The random allocation sequence was planned by one of the researchers (L.R.), and the participants’ enrollments were carried out by F.B., F.F., and L.E. Both the researchers (gastroenterologists and trained nutritionists) and the patients were blind after the assignment to each intervention group.

### 2.1. Clinical Evaluation

At the baseline and at the end of the intervention period, each patient underwent a clinical and nutritional evaluation (by two gastroenterologists and two trained nutritionists). Overall health and gastrointestinal and extra-gastrointestinal symptoms were assessed, and the gastrointestinal symptomatology was further classified. 

### 2.2. Diets 

The nutritional evaluation aimed at assessing anthropometrical parameters, nutritional status, and usual dietary patterns. After clinical evaluation, a personalized GFD adjusted to match energy and macronutrients and micronutrients daily requirements was indicated to each patient. This task was carried out by a trained nutritionist who was only in charge of performing this task, without involvement in patient management. In each dietary treatment, a structured 21-day dietary plan excluding all food gluten sources was indicated. The dietary plan included structured daily meals and specific foods/beverages and was explained in detail to each patient at the beginning of the study. After the initial explanation, the nutritionist was available to answer any doubts or issues strictly related to the dietary plan via e-mail or telephone. As all the patients received a structured dietary plan, both the R-GFD, as well as the LF-GFD, received a review of their current dietary habits, in addition to the change in FODMAP content in the LF-GFD group. The FODMAP content of the R-GFD and LF-GFD was a median (interquartile range) of 21.8 (18.5–22.5) and 3.7 (3.0–4.12) g/day, respectively, as previously described [27,28]. An example of the meals and foods used in both types of diets is shown in Table 1. The group with the LF-GFD received an in-depth GFD review, food education regarding GFD and LFD, and dietary counseling to initiate the modification of the FODMAP content towards the LFD. The patients in the R-GFD group received an in-depth GFD review together with food education regarding their diet. Compliance and doubts about the diet were checked 10 days later by means of a telephone call by the same nutritionist. 

### 2.3. Psychological Symptoms and Quality of Life

The Symptom Checklist-90-R (SCL-90) questionnaire was used to evaluate a broad range of psychological problems and symptomatology [29]. The scale is composed of 90 questions and assesses the presence and severity of symptoms of mental distress regarding different symptomatic domains (somatization, obsessive-compulsive, interpersonal sensitivity, depression, anxiety, hostility, phobic anxiety, paranoid ideation, and psychotic). Each question is awarded a score on a five-point Likert scale with extremes from “not at all” (0 points) to “extremely” (4 points). In addition to the scores rating specific symptoms’ intensities, a global severity index was calculated to estimate the assessment of the patient’s psychopathological state and as an indicator of symptomatic severity and psychic distress. 

The Short Form (36) Health Survey (SF-36) questionnaire evaluated the patients’ quality of life. This is a 36-question-long instrument conceptually referring to eight health domains: physical activity (10 questions), role limitations due to physical health (4 questions), role limitations due to emotional state (3 questions), physical pain (2 questions), general health perception (5 questions), vitality (4 questions), social activities (2 questions), mental health (5 questions), and a single question on changes in the state of health [30]. The scores for each domain ranged between 0 and 100, where 100 represents the best possible perception of quality of life. 

### 2.4. Gastrointestinal Symptoms

We used a series of 10-cm long visual analogue scales (VASs) referring to the level of satisfaction with their health status and the severity of specific symptoms (abdominal pain, satisfaction with stool consistency, bloating, postprandial fullness, early satiety, epigastric pain, and other symptoms). A further VAS evaluated satisfaction with general well-being (0 being extremely poor satisfaction and 10 very high satisfaction). These VASs were previously used by our group in a population with Non Celiac Gluten Sensitivity to evaluate gastrointestinal manifestations and general well-being [31]. The magnitude of change in gastrointestinal symptoms between the baseline and at the end of the 21-day-long intervention was assessed as follows: (a) comparing each VAS score at both time points, and (b) estimating the number of patients achieving a change in VAS score for general well-being higher than or equal to 50% from the baseline.

### 2.5. Statistical Analysis

The data were described as median ± Standard Deviation (SD) or median (inter-quartile range), depending on the parametric or non-parametric distribution of variables as assessed by graphical inspection and the Shapiro–Wilk test. All the patients who had fully completed the intervention period, were included in the analysis (per-protocol analysis). For SCL-90 and SF-36 scores, a within-group comparison at both time points (the baseline and day 21) and a between-group comparison at day 21 was conducted using an independent Student’s *t*-test or the non-parametric Wilcoxon rank sum test, depending on the distribution of variables. For the main outcome, the VAS score for general well-being, with two-way Analysis of Variance (ANOVA) (factors ‘treatment’ and ‘time’) with one repeated measure (‘time’), was used. The magnitude of change in the VAS score for the general well-being comparison between groups at day 21 was evaluated by Fisher’s exact two-tailed test. A 5% significance level was used, and the software packages STATA^®^ v. 13.1 (StataCorp LLC, College Station, TX, USA) and GraphPad Prism v. 6 (GraphPad Software, La Jolla, CA, USA) were used for analysis and figures processing.

## 3. Results

### 3.1. Patients

**Figure A1:**
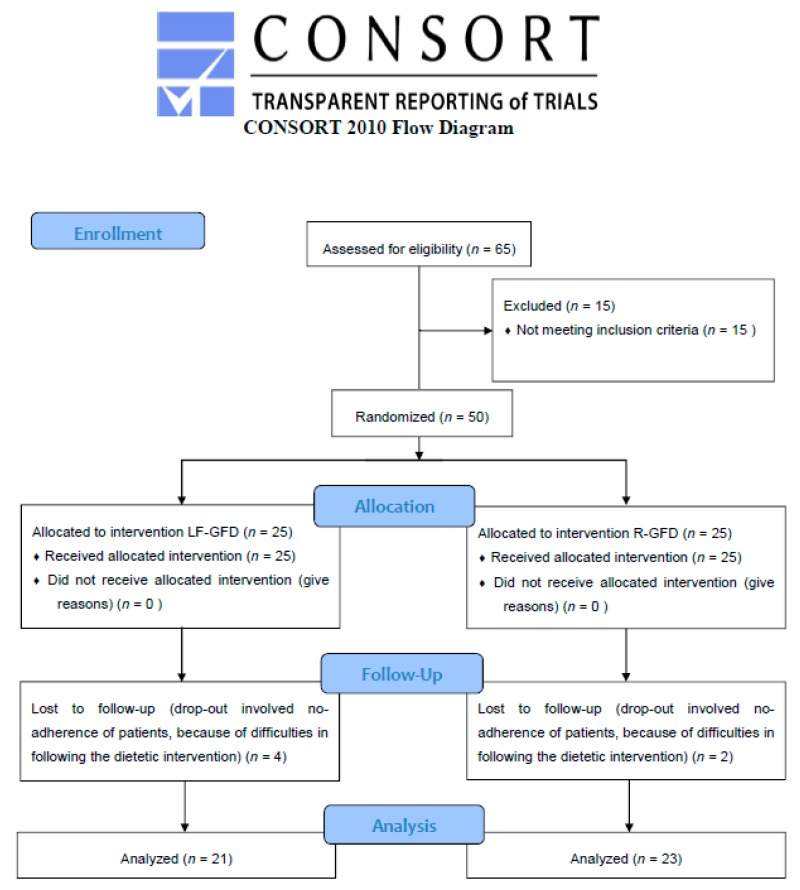
CONSORT Flow diagram.

The participant flow is shown in Figure A1. The patients were middle-aged, mainly women, and within the normal weight range, according to mean body-mass index (Table 2). Regarding their clinical symptomatology at the baseline, 64% reported the presence of IBS-like symptoms, whereas 34% reported functional symptomatology (Table 2). Among the specific symptoms, diarrhea and constipation were the most frequently reported (34% and 32%, respectively) with lower frequencies of mixed and non-specified gastrointestinal symptoms (12% and 8%, respectively). At the baseline the LF-GFD and R-GFD groups were similar in relation to the presence of evaluated symptoms.

### 3.2. Psychological Symptoms and Quality of Life

A consistent reduction in most SCL-90 scores was found in the LF-GFD group but not in the R-GFD group, with the global SCL-90 score being significantly reduced (*p* < 0.0003) compared with the R-GFD group at day 21 (*p* < 0.04, Table 3, and Figure S1 in the Supplementary Materials). With respect to specific sub-items of the SCL-90, there were no differences in the R-GFD group. However, in the LF-GFD group some significant changes were observed between the baseline and day 21 in relation to the majority (7 out of 9) of the psychopathological dimensions (Table 3). 

**Figure 1:**
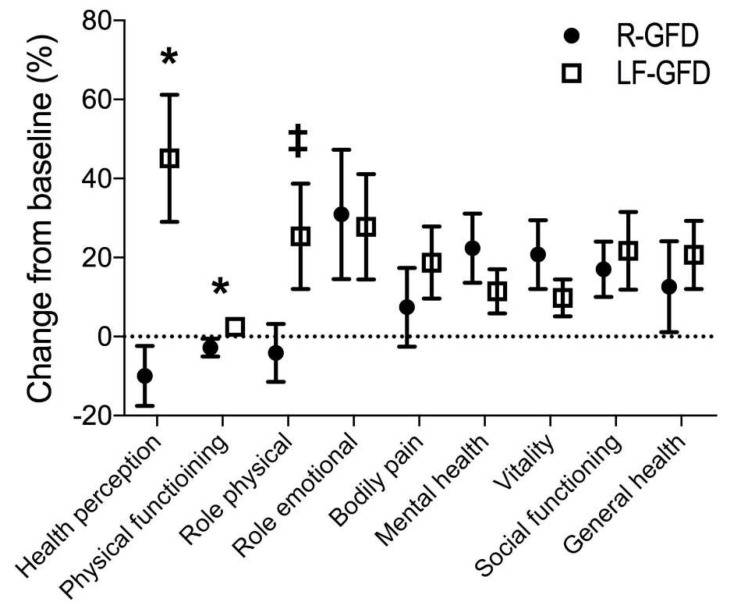
Change in the SF-36 questionnaire scores between the baseline and day 21 from intervention. Data shown as mean (symbol) ± SEM (upper and lower whiskers). For each sub-item, the difference between the values after intervention and the baseline was calculated and divided by the respective baseline value, expressed as a percentage, * *p* < 0.05; ^‡^ *p* = 0.06, for comparison between groups for each sub-item. R-GFD: regular gluten-free diet; and LF-GFD: low-FODMAP gluten-free diet.

The results of the SF-36 scores are shown in Table 4. Overall, there were no differences in the SF-36 sub-scores both within and between groups through the intervention (Table 4 and Figure S2 in the Supplementary Materials). However, when evaluating the change percentage at day 21, a statistically significant improvement in health perception, as well as in the physical functioning scores was found in the LF-GFD compared with the R-GFD group (Figure 1).

### 3.3. Gastrointestinal Symptoms

**Figure 2:**
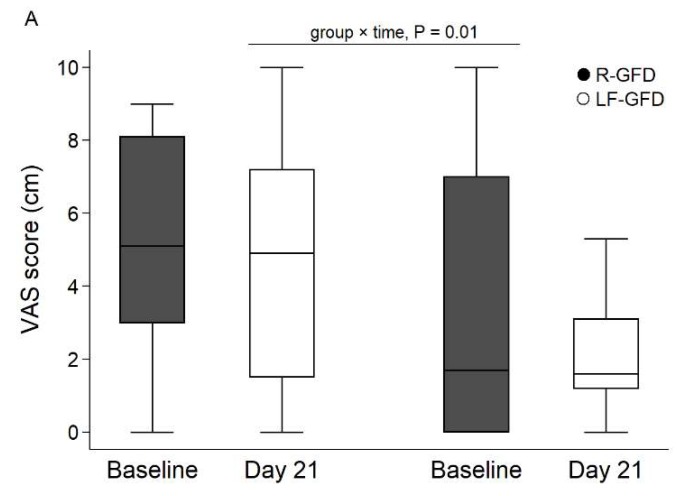

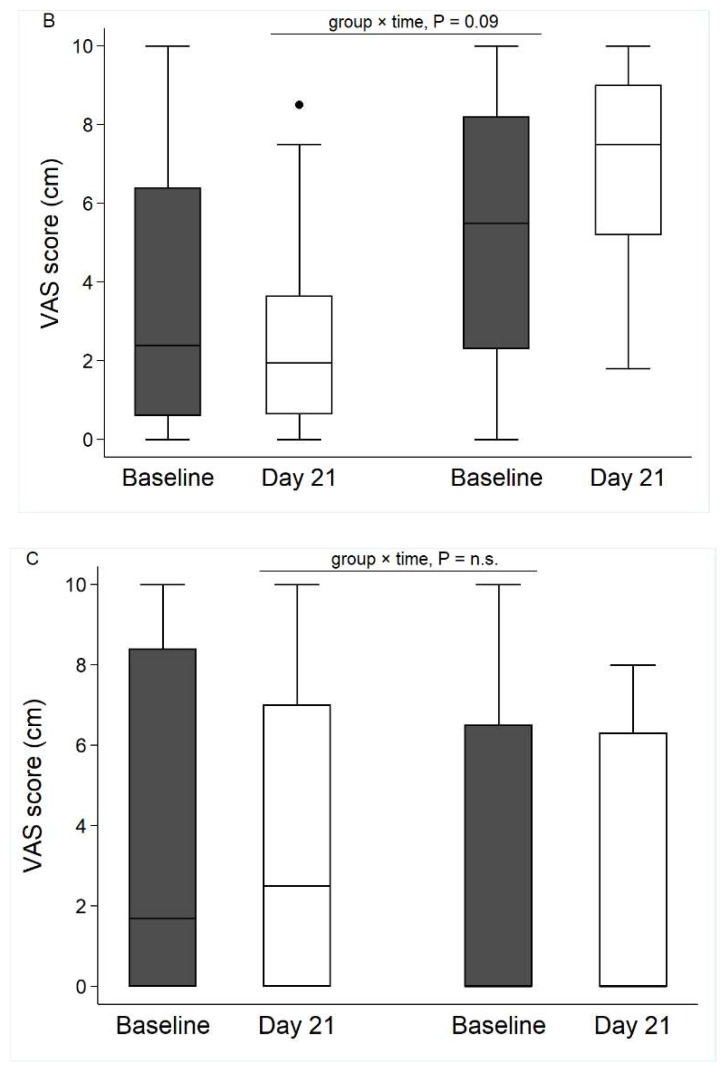
Visual analogue scale (VAS) score for gastrointestinal symptoms. VAS for abdominal pain (**A**), fecal consistency (**B**), and post-prandial fullness severity (**C**). In each plot, data is shown as median (line), inter-quartile range (box limits), and min/max (whiskers); Black dot in B) indicates an extreme value. R-GFD: regular gluten-free diet; LF-GFD: low-FODMAP gluten-free diet; and n.s.: non-significant.

A significant interaction was found with regard to the VAS score of abdominal pain with a significant decrease in the LF-GFD group versus the R-GFD group at day 21 (*p* < 0.01, Figure 2, and Figure S3 in the Supplementary Materials). The VAS score for satisfaction about fecal consistency showed a tendency for a higher increase in the LF-GFD group at day 21 (*p* < 0.09). Post-prandial fullness severity was lower in the LF-GFD group and decreased in both groups at day 21 (*p* < 0.006) but without significant interaction (Figure 2 and Figures S4 and S5 in the Supplementary Materials). No differences were found for non-specific functional gastrointestinal symptoms.

**Figure 3:**
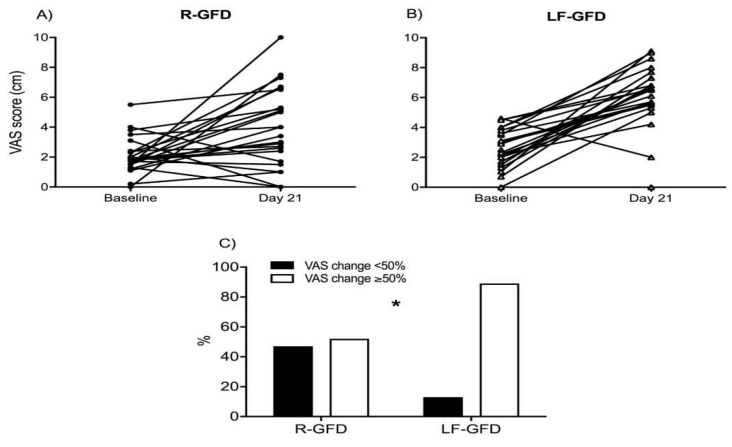
Change in the VAS of well-being between the baseline and day 21 from intervention. VAS score for overall well-being was evaluated at the baseline and at the end of the intervention period (day 21) for the R-GFD (**A**) and LF-GFD (**B**) groups; the magnitude of change in well-being perception (**C**) was calculated by estimating the number of patients (shown in percentage for each group) with a change in VAS score greater than or equal to 50% of the baseline value. Data are individual values at both time points. R-GFD: regular gluten-free diet; LF-GFD: low-FODMAP gluten-free diet; VAS: visual analogue scale. * *p* = 0.03 Fisher´s exact test.

The VAS score for satisfaction about general well-being was significantly enhanced in both groups. However, the improvement in well-being was greater in the LF-GFD group (*p* < 0.01, Figure 3). Consistently, the evaluation of the change in this VAS score of general well-being from the baseline revealed a greater change at day 21 in the LF-GFD group as compared with the R-GFD group (Figure 3).

## 4. Discussion

To our knowledge this is the first randomized double-blind intervention-controlled study that has investigated the effects of a LFD on patients with CD following GFDs but with persisting functional gastrointestinal symptoms. Our results showed a positive response to LFD, an improvement in psychological health scores—but only a limited change in quality of life—and a significant improvement in gastrointestinal symptoms with improved perception of well-being by the patients on the LFD.

Foods containing FODMAP can trigger IBS-like symptoms. These dietary compounds can trigger an increase in flatulence, diarrhea, and bloating that may lead to abdominal pain [13]. Our results suggest that LFD may improve persistent gastrointestinal symptomatology in those patients who undergo GFD and also successfully improve the psychological aspects already described in this group of patients [32]. After our intervention, the severity of gastrointestinal symptoms, such as abdominal pain and stool consistency, decreased when compared with the situation at the baseline and with the R-GFD group, along with improvement in the general well-being VAS. These results are in agreement with those reported by Halmos and colleagues, who showed that a low-FODMAP diet effectively reduced functional gastrointestinal symptoms in patients with IBS [17]. On the other hand, a study conducted on patients with inflammatory bowel disease has showed a positive response to LFD, thus suggesting that a reduction in FODMAP intake offers an efficacious strategy for those patients who present with concurrent functional gastrointestinal symptoms [33]. Although we were able to show an effect on symptomatology as measured by VAS in the LF-GFD group, we also observed that the patients receiving GFD reinforcement (our comparison group) did also show a decrease in symptomatology. This issue has already been addressed in Sainsbury and co-workers, [10], who emphasized that in-depth dietary revision as carried out by a trained nutritionist can improve any persistent symptomatology in CD patients. Nevertheless, our results have showed that such improvement was to a greater extent in the LF-GFD group.

Together with the improvements in IBS-like symptoms, we have also found that LF-GFD can overall improve the psychopathological symptoms as measured by a well-validated instrument. The relationship between psychological and psychiatric disturbances and CD is already well-established, significantly influencing the reduction of the quality of life and worsening the symptoms of affected patients [34]. The results related to the SCL-90 questionnaire have shown post-intervention differences in the LF-GFD group but none for the patients on the R-GFD. In the former group, in fact, we have observed a change in the vast majority of the dimensions as compared with the baseline, suggesting that the decrease in gastrointestinal symptoms may positively influence the improvement of a patient’s overall psychopathological burden. These results are consistent with those reported by a previous study, in which patients with CD undertook GFDs and for whom a post-intervention change was observed, which determined a decrease in the score for anxiety but not for depression [32].

In another report some patients newly diagnosed with CD were evaluated in relation to the dimensions of SCL-90 and compared with a healthy control group: the scores for somatization, obsessive-compulsive, interpersonal sensitivity, depression, anxiety, and sleep were found to be higher in CD patients [8]. The use of SCL-90 in a population that suffers from gastrointestinal symptoms has been already evaluated in IBS patients, who exhibited significantly more distress compared with other groups. In addition, the patients with gastrointestinal symptoms as a group, compared with the healthy controls, were characterized by high levels of irritable depression and somatization [35]. From this perspective, the results from the present study, which shows a significant psychopathological improvement in CD patients on the LF-GFD versus the R-GFD, provide an important confirmation about the relationship between gastrointestinal and mental health in CD patients who undergo different types of diet. In particular, to our knowledge this present study offers the first report demonstrating a significant amelioration for CD patients of most SCL-90 items in the short-term (i.e., after 21 days) as a result of following a LF-GFD. 

The quality-of-life perception, as assessed by means of the SF-36 questionnaire, has shown only minor differences between the studied groups: we could not establish for this group of celiac patients any significant improvement in this regard after the LFD. This finding is in discordance with what was previously reported. Previous data about our group of patients with non-celiac gluten sensitivity treated with GFDs, showed an improvement in the majority of the SF-36 scores after 7 days of treatment in a cross-over study, with both mental and physical components of the SF-36 questionnaire being significantly lower for patients positive to a gluten challenge [31]. Other authors have shown a quality-of-life improvement in patients with atypical and typical CD, compared with healthy controls after a one-year-long treatment, but only with differences in two items (general health and vitality) for subjects with typical CD [36].

Of note, even though we were not able to demonstrate any improvement in quality of life when comparing our study groups, when the percent change was evaluated for each of the items consulted, both health perception and physical functioning turned out higher in the LF-GFD group compared with the R-GFD group. Therefore, we could not rule out that the aforementioned mixed results possibly depended on the limited period of observation, with a longer follow-up period to be required in order to better assess the changes in quality of life for CD patients following the LF-GFD. 

Among the strengths of our report there is the fact that it comes from the first randomized double-blind study performed on patients with CD and to evaluate the potential effects of a reduction in dietary FODMAP on overall health and gastrointestinal symptomatology. As a limitation, even if we could show significant improvement in clinical symptoms, our results were obtained only after a short period of time (i.e., limited to three weeks) and only on patients who had fully completed the intervention (92% and 84% in the R-GFD and LF-GFD groups, respectively).

In conclusion, our results show that nutritional intervention by a LFD can have beneficial effects for CD patients who are on a GFD but present with persisting functional gastrointestinal disorders, even without major changes in their quality-of-life perception. The same results also suggest that, for those patients with CD being treated with a GFD and experiencing IBS-like symptoms, a LFD can be indicated by a trained nutritionist, but its beneficial effects and long-term clinical effects for this group of selected CD patients need further investigation.

ClinicalTrials.gov, ref. no. DNCT02946827.

## Figures and Tables

**Table 1:** Examples of the two different prescribed diets for a typical day ^1^.

Meal	LF-GFD (3.54 g/day FODMAP)	R-GFD (19.9 g/day FODMAP)
Breakfast	1 cup of tea 80 g of gluten free biscuits	1 glass of fresh orange juice 3 slices of gluten-free bread 3 teaspoons of honey
Morning snack	1 banana	1 apple
Lunch	130 g of gluten free pasta with zucchini 60 g of chicken 150 g of carrots	120 g of turkey thighs 200 g of cauliflowers
Afternoon snack	1 cup of blueberries	1 pear
Dinner	180 g of seafood 150 g of tomatoes	200 g of asparagus soup 70 g of fresh cheese 150 g of carrots
During the day	130 g of gluten free bread 6 tea spoons and half of virgin olive oil	160 g of gluten free bread 2 tea spoons of virgin olive oil

^1^ Dietary data represent the typical diet for a patient with an approximate energy expenditure of 1800 kcal/day. FODMAP: Fermentable, oligosaccharides, disaccharides, monosaccharides, and polyols; R-GFD: regular gluten-free diet; and LF-GFD: low-FODMAP diet.

**Table 2:** Background and gastrointestinal symptoms at baseline ^1^.

Variable	Overall (*n* = 50)	R-GFD (*n* = 25)	LF-GFD (*n* = 25)	*p* value ^†^
Age, years	41.1 ± 10.1	40.4 ± 10.1	41.9 ± 10.2	0.73
Gender, female (%)	44 (88)	25 (100)	22 (3 no data)	0.09
BMI, kg/m^2^	22.5 ± 4.1	22.3 ± 3.6	22.1 ± 5.4	0.87
Diarrhea, *n* (%)	17 (34)	6 (6 no data)	11 (4 no data)	0.18
Constipation, *n* (%)	16 (32)	9 (7 no data)	7 (2 no data)	0.2
Mixed symptoms, *n* (%)	6 (12)	4 (10 no data)	2 (5 no data)	0.36
Non-specified, *n* (%)	4 (8)	3 (12 no data)	1 (9 no data)	0.29
Dyspepsia, *n* (%)	17 (34)	8 (5 no data)	9 (3 no data)	0.95

^1^ Data shown as mean ± Standard Deviation (SD) for continuous variables and frequency and percentage for nominal variables. ^†^ *p*-value for comparison between groups using an independent *t*-test for continuous variables or Chi-square or Fisher’s exact tests for nominal variables. BMI: body-mass index; R-GFD: regular gluten-free diet; and LF-GFD: low-FODMAP gluten-free diet.

**Table 3:** SCL-90 scores according to studied groups ^1^.

	R-GFD			LF-GFD			
	Baseline (*n* = 25)	21-day (*n* = 23)	*p*-Value within Group ^†^	Baseline (*n* = 25)	21-day (*n* = 21)	*p*-Value within Group ^†^	*p*-Value between Groups
Global index	1.61 ± 0.39	1.44 ± 0.29	0.13 ^‡^	1.49 ± 0.17	1.26 ± 0.18	0.0003	0.04
Somatization	1.87 (0.71)	1.50 (0.58)	0.13	1.83 (0.62)	1.45 (0.40)	0.01	0.43
Obsessive-compulsive	1.70 (1.0)	1.60 (0.70)	0.41	1.60 (0.50)	1.30 (0.60)	0.01	0.15
Interpersonal sensitivity	1.55 (0.66)	1.38 (0.65)	0.09	1.22 (0.44)	1.11 (0.44)	0.09	0.58
Depression	1.57 (0.73)	1.50 (0.73)	0.33	1.54 (0.46)	1.38 (0.46)	0.01	0.26
Anxiety	1.30 (0.65)	1.10 (0.51)	0.17	1.30 (0.40)	1.10 (0.15)	0.02	0.60
Hostility	1.42 (0.50)	1.42 (0.58)	0.75	1.33 (0.50)	1.16 (0.08)	0.01	0.11
Phobic anxiety	1.0 (0.14)	1.0 (0.14)	0.81	1.0 (0.14)	1.0 (0.0)	0.10	0.12
Paranoid ideation	1.66 (0.83)	1.16 (0.66)	0.22	1.33 (0.50)	1.0 (0.33)	0.01	0.20
Psychotic	1.20 (0.50)	1.10 (0.25)	0.13	1.20 (0.10)	1.0 (0.10)	0.03	0.26

^1^ Data shown as mean ± SD or median (interquartile range) for non-parametrical variables. ^†^ *p*-value for comparison within groups using a non-parametric Wilcoxon rank sum test unless otherwise is indicated; ^‡^ independent *t*-test. R-GFD: regular gluten-free diet; LF-GFD: low-FODMAP gluten-free diet.

**Table 4:** Short Form (36) Health Survey (SF-36) subscales and global score ^1^.

	R-GFD			LF-GFD			
	Baseline (*n* = 25)	21-day (*n* = 23)	*p*-value within Group ^†^	Baseline (*n* = 25)	21-day (*n* = 21)	*p*-value within Group ^†^	*p*-value between Groups
Health perception	46.44 ± 29.62	52.0 ± 26.93	0.53	44.54 ± 24.25	53.27 ± 18.92	0.17	0.87
Physical functioning	95.0 (15.0)	100 (30)	0.94 ^‡^	95.0 (7.5)	95.0 (10.0)	0.25	0.91
Role physical	75.0 ± 34.6	81.3 ± 32.2	0.40 ^‡^	79.2 ± 28.2	87.5 ± 26.4	0.18	0.65
Role emotional	69.3 ± 35.9	77.1 ± 35.9	0.41 ^‡^	65.3 ± 37.4	81.8 ± 28.6	0.10	0.82
Bodily Pain	60.88 ± 23.90	64.93 ± 26.37	0.62	65.91 ± 19.29	72.77 ± 20.54	0.25	0.33
Mental health	63.91 ± 19.47	69.33 ± 14.78	0.33	62.38 ± 16.92	66.18 ± 13.89	0.40	0.51
Vitality	50.20 ± 18.14	59.66 ± 19.77	0.14	55.20 ± 14.02	57.27 ± 12.41	0.59	0.68
Social functioning	69.0 ± 22.27	78.33 ± 20.30	0.18	69.27 ± 18.78	76.13 ± 17.63	0.20	0.73
General health	53.25 ± 26.12	62.26 ± 25.28	0.29	57.47 ± 18.91	62.36 ± 17.08	0.36	0.98

^1^ Data shown as mean ± SD or median (interquartile range) for non-parametrical variables. ^†^ *p*-value for comparison within groups using an independent *t*-test unless otherwise is indicated; ^‡^ non-parametric Wilcoxon rank sum test. R-GFD: regular gluten-free diet; LF-GFD: low-FODMAP gluten-free diet.

## Supplementary Materials

The following are available online at http://www.mdpi.com/2072-6643/10/8/1023/s1, Figure S1: SCL-90 global index, Figure S2: SF-36 score for general health, Figure S3: VAS score for abdominal pain, Figure S4: VAS score for fecal consistency, Figure S5: VAS score for postprandial fullness severity.
