# Correction: Roncoroni, L. et al. A Low FODMAP Gluten-Free Diet Improves Functional Gastrointestinal Disorders and Overall Mental Health of Celiac Disease Patients: A Randomized Controlled Trial. *Nutrients* 2018, *10*, 1023

**DOI:** 10.3390/nu11030566

**Published:** 2019-03-06

**Authors:** Leda Roncoroni, Karla A. Bascuñán, Luisa Doneda, Alice Scricciolo, Vincenza Lombardo, Federica Branchi, Francesca Ferretti, Bernardo Dell’Osso, Valeria Montanari, Maria Teresa Bardella, Luca Elli

**Affiliations:** 1 Centre for the Prevention and Diagnosis of Celiac Disease/Division of Gastroenterology and Endoscopy, Fondazione IRCCS Ca’ Granda Ospedale Maggiore Policlinico, 20122 Milan, Italy; kbascunan@med.uchile.cl (K.A.B.); scricciolo.alice@gmail.com (A.S.); vicky.l@hotmail.it (V.L.); federica.branchi@gmail.com (F.B.); francesca.ferretti01@gmail.com (F.F.); montanari.valeria8@gmail.com (V.M.); mariateresa.bardella@yahoo.com (M.T.B.); luca.elli@policlinico.mi.it (L.E.); 2 Department of Biomedical, Surgical and Dental Sciences, University of Milano, 20100 Milan, Italy; luisa.doneda@unimi.it; 3 Department of Nutrition, University of Chile, 8380453 Santiago, Chile; 4 Department of Pathophysiology and Transplantation, Università degli Studi di Milano, Fondazione IRCCS Ca’ Granda, Ospedale Maggiore Policlinico, 20100 Milan, Italy; bernardo.dellosso@unimi.it; 5 Department of Psychiatry and Behavioral Sciences, Bipolar Disorders Clinic, Stanford University, Stanford, CA 94305-5723, USA; 6 CRC “Aldo Ravelli” for Neuro-technology & Experimental Brain Therapeutics, University of Milan, 20100 Milan, Italy

The authors have requested that the following changes be made to their paper [1].

In Table 2, page 6, the frequency (and percentage) of gastrointestinal symptoms are shown for each group (regular gluten-free diet (R-GFD) and low fermentable, oligosaccharides, disaccharides, monosaccharides, and polyols (FODMAP)—gluten free diet (LF-GFD)). Instead of showing the percentages for each group, "No data" was included in parentheses instead of the percentages corresponding to the frequency of symptoms in each group. The table should read as follows.

In Figure 2, page 8, visual analogue scale scores for abdominal pain, fecal consistency, and post-prandial fullness severity are shown. Instead of stating only both times of assessment (at “baseline” and “day 21”), a duplicate in the writing of the *x*-axis legend was found. The figure should read as follows.

The authors apologize to the readers for any inconvenience caused by these changes. It is important to state that both corrections do not affect our study’s results and involve no changes or modifications in the original data supporting our results. The original manuscript will remain online on the article webpage, with reference to this Correction.

## Figures and Tables

**Figure 2:**
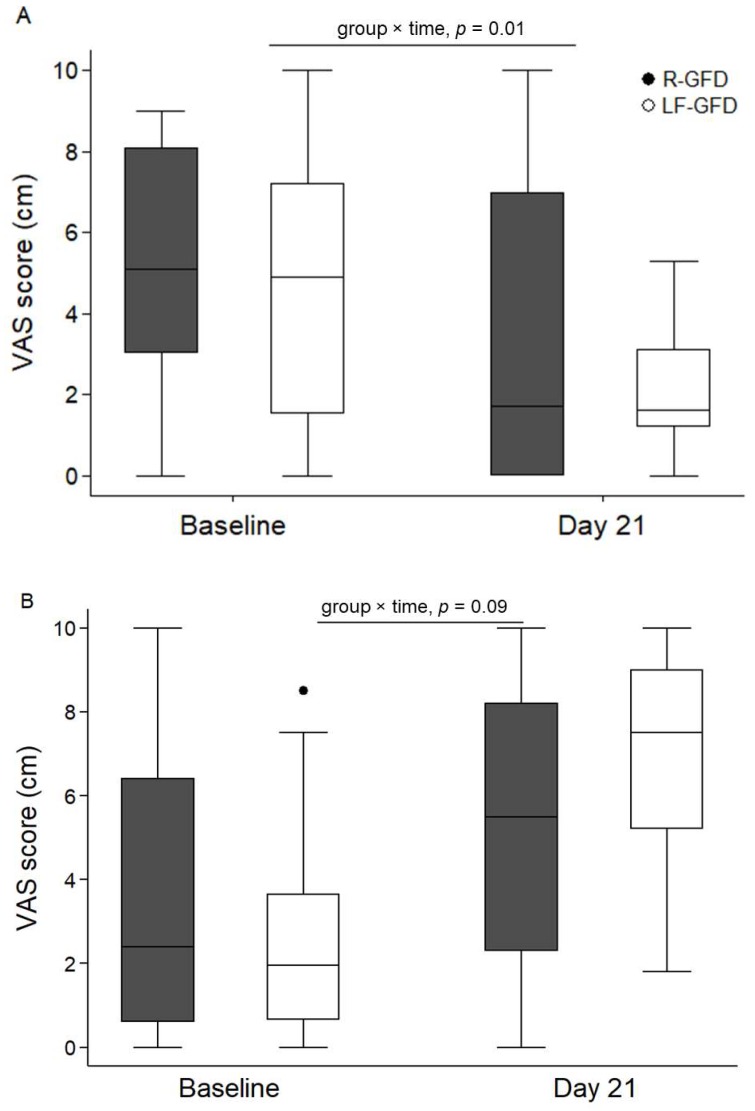

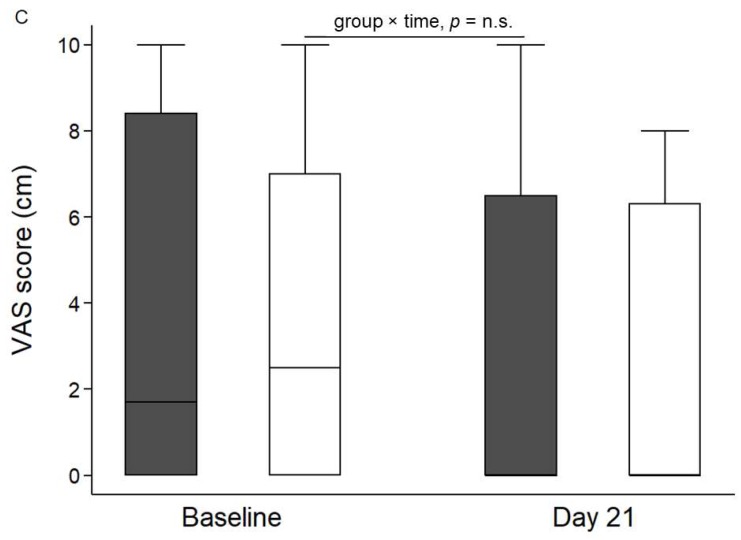
Visual analogue scale (VAS) score for gastrointestinal symptoms. VAS for abdominal pain (**A**), fecal consistency (**B**), and post-prandial fullness severity (**C**). In each plot, data is shown as median (line), inter-quartile range (box limits), and min/max (whiskers); R-GFD: regular gluten-free diet; LF-GFD: low-FODMAP gluten-free diet; n.s.: non-significant.

**Table 2:** Background and gastrointestinal symptoms at baseline ^1^.

Variable	Overall (*n* = 50)	R-GFD (*n* = 25)	LF-GFD (*n* = 25)	*p* Value ^†^
Age, years	41.1 ± 10.1	40.4 ± 10.1	41.9 ± 10.2	0.73
Gender, female (%)	44 (88)	25 (100)	22 (88)	0.09
BMI, kg/m^2^	22.5 ± 4.1	22.3 ± 3.6	22.1 ± 5.4	0.87
Diarrhea, *n* (%)	17 (34)	6 (24)	11 (44)	0.18
Constipation, *n* (%)	16 (32)	9 (36)	7 (28)	0.2
Mixed symptoms, *n* (%)	6 (12)	4 (16)	2 (8)	0.36
Non-specified, *n* (%)	4 (8)	3 (12)	1 (4)	0.29
Dyspepsia, *n* (%)	17 (34)	8 (32)	9 (36)	0.95

^1^ Data shown as mean ± standard deviation (SD) for continuous variables and frequency and percentage for nominal variables. ^†^ *p*-value for comparison between groups using an independent *t*-test for continuous variables or Chi-square or Fisher’s exact tests for nominal variables. BMI: body mass index; R-GFD: regular gluten-free diet; LF-GFD: low fermentable, oligosaccharides, disaccharides, monosaccharides, and polyols (FODMAP)—gluten free diet (LF-GFD).

## References

[1] Roncoroni L., Bascuñán K.A., Doneda L., Scricciolo A., Lombardo V., Branchi F., Ferretti F., Dell’osso B., Montanari V., Bardella M.T. (2018). A low FODMAP gluten-free diet improves functional gastrointestinal disorders and overall mental health of celiac disease patients: A randomized controlled trial. Nutrients.

