# Supplementary material for: A Low FODMAP Gluten-Free Diet Improves Functional Gastrointestinal Disorders and Overall Mental Health of Celiac Disease Patients: A Randomized Controlled Trial
Source: Nutrients. 2018 Aug 4;10(8):1023. doi: 10.3390/nu10081023 (PMC6115770; doi:10.3390/nu10081023)
Supplement: Supplementary file 1 [file nutrients-10-01023-s001.pdf]

Supplementary Materials

Figure S1. SCL-90 global index

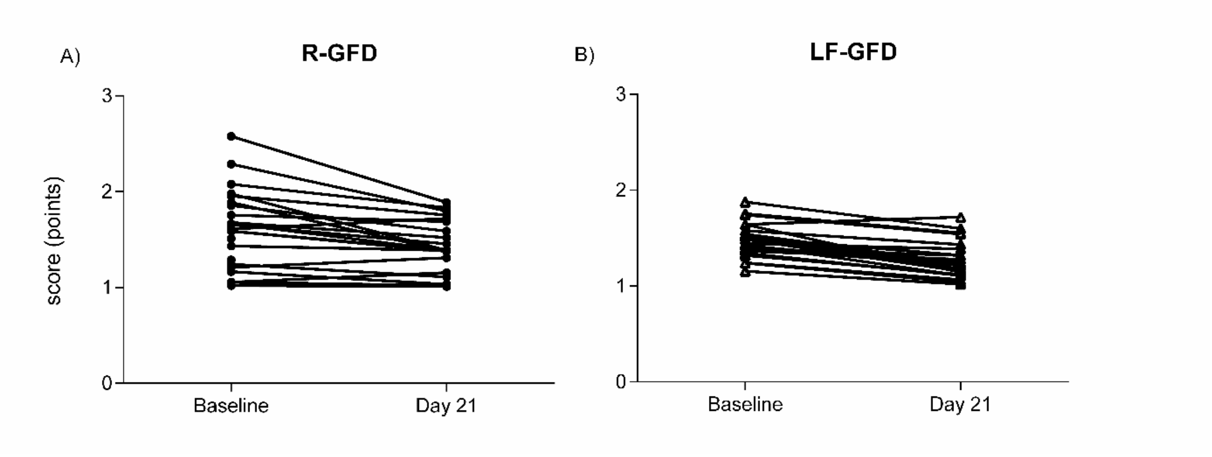

Changes in the SCL-90 global index score for score for psychological symptoms between the baseline and day 21 from the intervention, evaluated at the baseline and at the end of the intervention period (day 21) for the R-GFD (A) and LF-GFD (B) groups. Data are individual values at both time points. SCL-90: Symptom Checklist-90-R (SCL-90); R-GFD: regular gluten-free diet; and LF-GFD: low-FODMAP gluten-free diet.

Figure S2. SF-36 score for general health

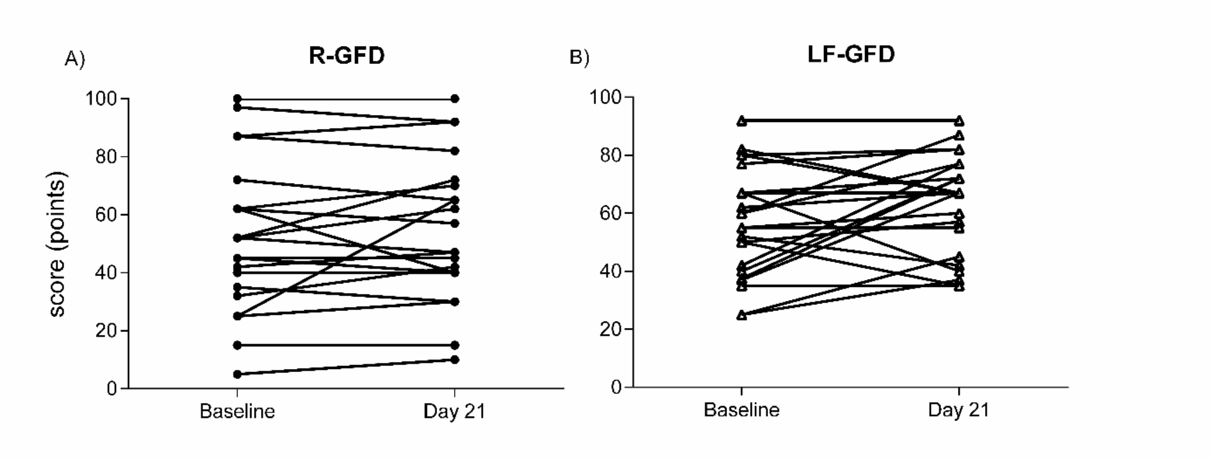

Changes in the SF-36 score for general health between the baseline and day 21 from the intervention, evaluated at the baseline and at the end of the intervention period (day 21) for the R-GFD (A) and LF-GFD (B) groups. Data are individual values at both time points. SF-36: Short Form (36) Health Survey questionnaire; R-GFD: regular gluten-free diet; LF-GFD: and low-FODMAP gluten-free diet.

Figure S3. VAS score for abdominal pain

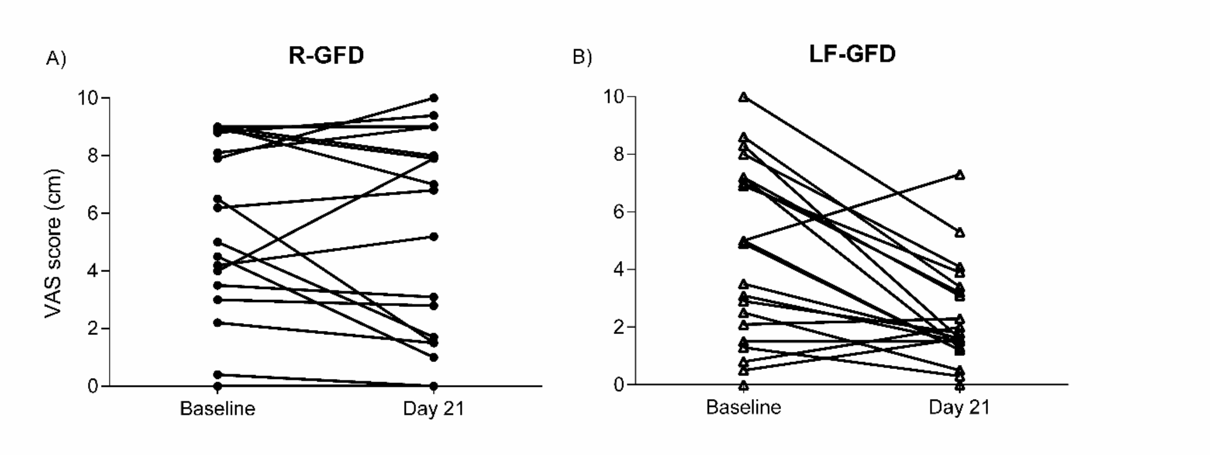

Change in the VAS score for abdominal pain between the baseline and day 21 from intervention. The VAS score for abdominal pain was evaluated at the baseline and at the end of the intervention period (day 21) for the R-GFD (A) and LF-GFD (B) groups. Data are individual values at both time points. R-GFD: regular gluten-free diet; LF-GFD: low-FODMAP gluten-free diet; and VAS: visual analogue scale.

Figure S4. VAS score for fecal consistency

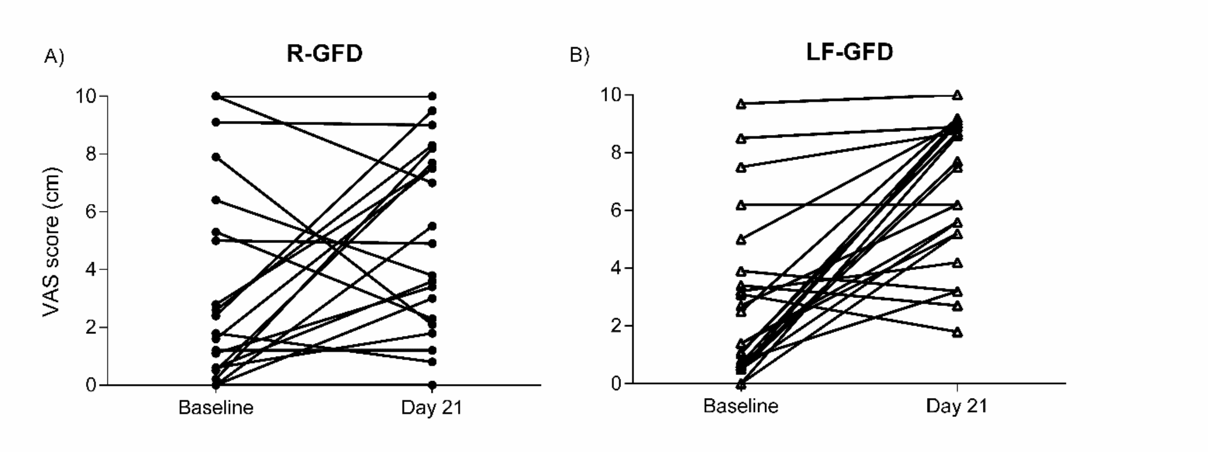

Change in the VAS score for fecal consistency between the baseline and day 21 from intervention. VAS score for abdominal pain was evaluated at the baseline and at the end of the intervention period (day 21) for the R-GFD (A) and LF-GFD (B) groups. Data are individual values at both time points. R-GFD: regular gluten-free diet; LF-GFD: low-FODMAP gluten-free diet; and VAS: visual analogue scale.

Figure S5. VAS score for postprandial fullness severity

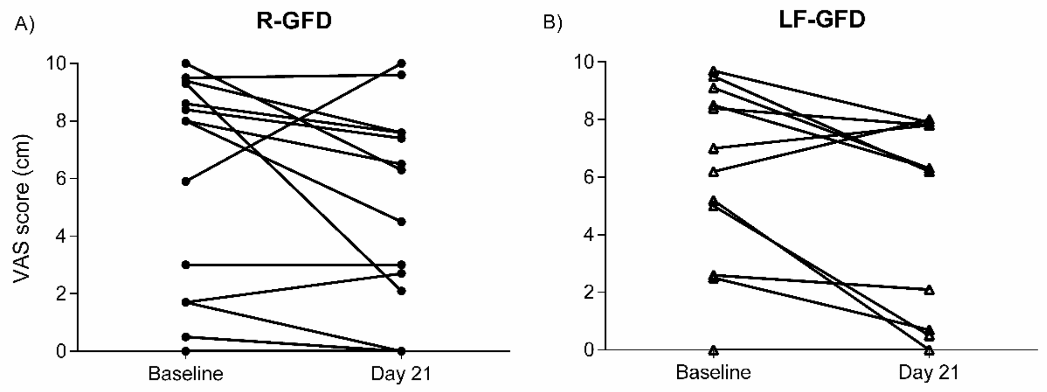

Change in the VAS score for postprandial fullness severity between the baseline and day 21 from the intervention. The VAS score for abdominal pain was evaluated at the baseline and at the end of the intervention period (day 21) for the R-GFD (A) and LF-GFD (B) groups. Data are individual values at both time points. R-GFD: regular gluten-free diet; LF-GFD: low-FODMAP gluten-free diet; and VAS: visual analogue scale.
